# Psychiatric History and Postpartum Depression: The Mediating Role of Obstetric Complications

**DOI:** 10.1111/acps.70105

**Published:** 2026-05-05

**Authors:** Mette‐Marie Zacher Kjeldsen, Katrine Holde, Line Bager, Veerle Bergink, Emely Ek Blæhr, Janne Tidselbak Larsen, Kathrine Bang Madsen, Merete Lund Mægbæk, Liselotte Vogdrup Petersen, Trine Munk‐Olsen

**Affiliations:** ^1^ National Centre for Register‐Based Research, Department of Public Health Aarhus University Aarhus Denmark; ^2^ CIRRAU—Centre for Integrated Register‐Based Research, Department of Public Health Aarhus University Aarhus Denmark; ^3^ Department of Psychiatry Icahn School of Medicine at Mount Sinai New York New York USA; ^4^ Department of Psychiatry Erasmus Medical Center Rotterdam the Netherlands; ^5^ Department of Clinical Research University of Southern Denmark Odense Denmark; ^6^ Research Unit of Child and Adolescent Psychiatry Mental Health Services in the Region of Southern Denmark Odense Denmark

**Keywords:** mediation analysis, obstetric complications, postpartum depression, psychiatric history

## Abstract

**Introduction:**

Psychiatric history is the strongest risk factor for postpartum depression (PPD). Obstetric complications, more prevalent among women with a psychiatric history, are also independent risk factors. However, the mechanisms linking these factors to PPD remain unclear. We examined whether obstetric complications mediate the association between psychiatric history and PPD.

**Methods:**

This cohort study utilized Danish nationwide Edinburgh Postnatal Depression Scale (EPDS) screenings (2015–2021) linked with register data. Psychiatric history was defined as psychiatric diagnoses (ICD‐10: F00‐99) or filled psychotropic prescriptions (ATC: N05A, N05BE01, N06A, N06BA) from 1995 until conception. Complications were defined as a composite measure of complications occurring between conception and delivery. *PPD symptoms* were defined as a positive EPDS score (≥ 11), and *PPD diagnosis* was defined as a depression diagnosis (ICD‐10: F32‐33) or antidepressant prescription fill (ATC: N06A indicated for depression).

**Results:**

Of 170,218 mothers (163,326 in analyses), 23.9% had a psychiatric history. These mothers had higher levels of PPD symptoms (13.4% vs. 6.1%), PPD diagnosis (7.0% vs. 0.4%), and complications (34.1% vs. 28.5%) compared to those without. (A) Psychiatric history (PPD symptoms: OR = 2.32 [95% CI, 2.22; 2.41]; PPD diagnosis: OR = 5.09 [95% CI, 4.48; 5.79]) and complications (PPD symptoms: OR = 1.16 [95% CI, 1.11; 1.21]; PPD diagnosis: OR = 1.18 [95% CI, 1.04; 1.34]) were independently associated with PPD. (B) Psychiatric history did not modify the association between complications and PPD (PPD symptoms: OR = 1.20 [95% CI, 1.14; 1.26] vs. 1.09 [95% CI, 1.02; 1.17]; PPD diagnosis: OR = 1.22 [95% CI, 1.00; 1.49] vs. 1.15 [95% CI, 0.97; 1.36]). (C) Complications mediated only a small fraction of the association between psychiatric history and PPD (proportion mediated: PPD symptoms = 0.68% [95% CI, 0.50%; 1.00%], PPD diagnosis = 0.42% [95% CI, 0.14%; 0.79%]).

**Conclusions:**

Psychiatric history and complications are independently associated with PPD, but complications explain only a negligible portion. These findings suggest that the link between psychiatric vulnerability and PPD is primarily driven by direct mechanisms rather than mediation through complications.

## Highlights

- Significant Outcomes
  ○ Personal psychiatric history and obstetric complications were independently associated with postpartum depression, with psychiatric history showing the strongest association.
  ○ No evidence was found for interaction between psychiatric history and obstetric complications.
  ○ Obstetric complications did not mediate the association between personal psychiatric history and postpartum depression, suggesting that these risk factors operate through distinct pathways.
- Limitations
  ○ The HOPE cohort underrepresents non‐Danish populations, limiting generalizability to immigrant populations.
  ○ Despite adjustment for measured sociodemographic and reproductive factors, residual confounding may remain (e.g., social support, unintended pregnancy, physical activity).

## 1 Introduction

Postpartum depression (PPD) affects one in six new mothers [1, 2]. Despite well‐documented risk factors, PPD remains underdiagnosed and undertreated [3, 4]. The complex interplay between risk factors likely contributes to challenges in identifying at‐risk women, underscoring the need for a deeper understanding of underlying disease mechanisms.

A personal psychiatric history is the strongest predictor of PPD [5, 6, 7, 8, 9, 10, 11]. More specifically, risk of PPD is elevated across a broad range of psychiatric disorders, including major depressive disorder (MDD), bipolar disorder (BD), anxiety and panic disorders, posttraumatic stress disorder (PTSD), eating disorders, attention deficit hyperactivity disorder (ADHD), and obsessive–compulsive disorder (OCD) [12, 13, 14, 15].

Importantly, women with a psychiatric history are also at heightened risk of obstetric complications, particularly when having recent or persistent psychiatric episodes [16, 17]. These obstetric complications, which are also independently associated with an increased PPD risk, include preeclampsia [18], gestational diabetes [9, 19], gestational hypertension [10], hyperemesis gravidarum [20], postpartum hemorrhage [21], Cesarean section (C‐section) [22], and preterm birth [23, 24]. However, whether these obstetric complications mediate the association between personal psychiatric history and PPD remains unexplored. Examining this provides important insights to uncover mechanisms underlying PPD and advancing personalized prevention and intervention.

### 1.1 Aims of the Study

This study aimed to estimate the extent to which obstetric complications mediate the association between personal psychiatric history and PPD. We hypothesized that these obstetric complications partially mediate the association, meaning women with a psychiatric history are at increased risk of PPD both directly and indirectly through a higher likelihood of experiencing obstetric complications during pregnancy and delivery.

## 2 Materials and Methods

This study followed the AGReMA guideline [25]. A preregistered protocol is available at *Open Science Framework* (https://osf.io/x85d9/).

### 2.1 Data Sources and Study Population

This cohort study utilized the HOPE cohort, comprising 170,218 maternal Edinburgh Postnatal Depression Scale (EPDS) screenings conducted within 12 weeks postpartum (January 1, 2015, to December 31, 2021), linked with Danish population‐wide registers [26, 27]. The cohort is broadly representative of the Danish background population; details are available elsewhere [26].

To ensure that the outcome, PPD, was not a continuation of a pre‐existing depression, a wash‐out period was implemented. All mothers with a recorded depression diagnosis (ICD‐10: F32‐33) or a redeemed prescription for antidepressants (ATC: N06A) from conception until delivery were excluded. Data on ICD‐10 diagnoses and ATC codes were retrieved from the Psychiatric Central Research Register, the National Patient Register, and the National Prescription Register [28, 29, 30]. See Figure S1 for details on variables and temporal order.

### 2.2 Exposure

Personal psychiatric history was the exposure of interest. It was defined as either a redeemed psychotropic prescription (ATC: N05A (antipsychotics), N05BE01 (buspirone), N06A (antidepressants), N06BA (psychostimulants)) or a psychiatric diagnosis (ICD‐10: F00‐99) recorded from 1995 until conception. It was categorized as “Yes” or “No” and sourced from the Psychiatric Central Research Register, the National Patient Register, and the National Prescription Register [28, 29, 30].

### 2.3 Outcome

PPD was the outcome of interest and was operationalized using two separate measures, both dichotomized (“Yes” or “No”):

1. *PPD symptoms* were defined as a positive EPDS screening (cut‐off ≥ 11) within 12 weeks postpartum [31].
2. *PPD diagnosis* was defined as a redeemed antidepressant prescription (ATC: N06A) with indication for depression (indication codes: 168 and 270), or an in‐ or outpatient hospital contact with a depression diagnosis (ICD‐10: F32‐33) within 6 months postpartum. Data were sourced from the National Prescription Register, the National Patient Register, and the Psychiatric Central Research Register [28, 29, 30].

Previous research has shown that the overlap between *PPD symptoms* and *PPD diagnosis* in the HOPE cohort is only 0.8%, indicating that these are two distinct definitions of PPD [26].

### 2.4 Mediators

Obstetric complications were the potential mediators of interest. Obstetric complications included preeclampsia/eclampsia (ICD‐10: O14‐15), gestational hypertension (ICD‐10: O13), gestational diabetes (ICD‐10: O24), hyperemesis gravidarum (ICD‐10: O21), postpartum hemorrhage (ICD‐10: O72 code VPH ≥ 1000) [32], acute C‐section (ICD‐10: DO821, DO843, DO829, DO842, procedure codes: KMCA10A and KMCA10E), and preterm birth (delivery < 37 weeks of gestation). Each obstetric complication was categorized as “Yes” or “No.” Based on all obstetric complications, a composite variable was created (“Any complications” or “No complications”). Data were collected from the Medical Birth Register and the National Patient Register [30, 33].

### 2.5 Covariates

As outlined in the Directed Acyclic Graph (DAG) (Figure S2), analyses were adjusted for potential confounders in the associations between exposure, mediator, and outcome. These included parity, sourced from the Medical Birth Register, categorized as “1 child,” “2 children,” or “3+ children*”* [33]. Calendar year and maternal age at delivery, sourced from the Civil Registration System, were included as continuous variables and modeled linearly [34]. Education level, sourced from Statistics Denmark's Register of Education, categorized as “mandatory” (primary education), “short” (upper secondary and vocational training), “medium” (bachelor's degree), or “high” (master's and doctoral degrees) [35, 36]. And lastly, maternal origin of birth, sourced from the Civil Registration System, categorized as “Denmark” or “Not Denmark” [34].

### 2.6 Statistical Analyses

Descriptive characteristics of the study population were stratified by exposure status, comparing mothers with and without psychiatric history.

**FIGURE 1:**
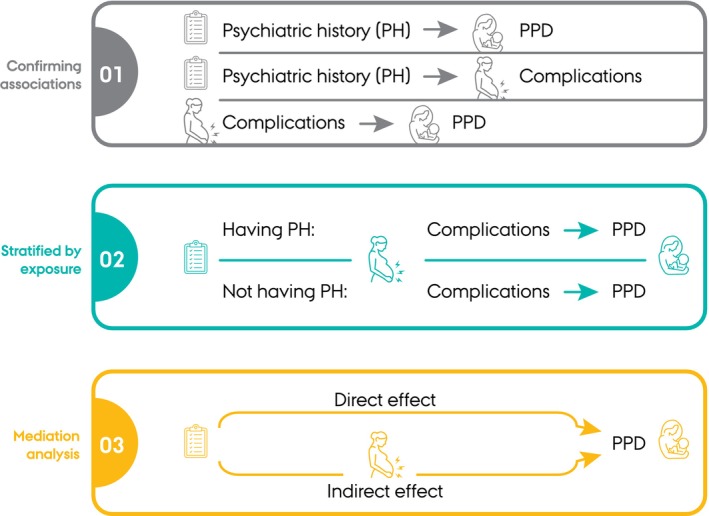
Analytical approach.

The statistical analyses followed a three‐step approach (Figure 1) using logistic regression models with robust standard errors to estimate odds ratios (OR) with 95% confidence intervals (CIs). Robust standard errors accounted for some mothers being included more than once in the HOPE cohort. *First*, associations between the exposure and outcome (adjusted for parity, calendar year, educational level, and origin of birth), the mediator and outcome (adjusted for personal psychiatric history, parity, calendar year, maternal age, educational level, origin of birth), and the exposure and mediator (adjusted for parity, calendar year, maternal age, educational level, and country of origin) were estimated to confirm independent associations. *Second*, the association between the mediator (obstetric complications) and outcome (PPD), stratified by the exposure (psychiatric history), was estimated using fully adjusted models. Due to minimal variation across strata, the mediation models were specified without an exposure‐mediator interaction term. *Third*, causal mediation analysis within the counterfactual framework was applied to examine the mediating role of obstetric complications in the association between psychiatric history and PPD [37]. This approach decomposed the *total effect* of psychiatric history on PPD into the *natural direct effect (NDE)*—the effect of psychiatric history on PPD independent of complications—and the *natural indirect effect (NIE)*—the effect mediated through obstetric complications. The *proportion mediated* was then estimated as the $\frac{\mathrm{NIE}}{\text{Total effect}}$[38]. The proportion mediated quantifies the proportion of the total effect explained by obstetric complications. The primary analysis focused on the mediator variable indicating the presence or absence of any obstetric complications, while individual obstetric complications were analyzed separately. A secondary mediation analysis stratified psychiatric history by timing: “≥ 3 years prior to conception” and “< 3 years prior to conception.” As missing data were < 5%, adjusted analyses were conducted using complete case analysis. CIs for the NDE and NIE were estimated using a quasi‐Bayesian approximation with 10 Monte Carlo draws.

Given the binary outcome, causal mediation analysis offers advantages over traditional methods due to its flexibility, of not being restricted to parametric models [39]. However, this approach comes with the following assumptions: (1) no unmeasured confounding of the exposure‐outcome relationship, (2) no unmeasured confounding of the mediator‐outcome relationship, (3) no unmeasured confounding of the exposure‐mediator relationship, and (4) no confounders of the mediator‐outcome effect that are affected by the exposure (cross‐world independence assumption) [39]. These assumptions were considered in the a priori DAG (Figure S2) and evaluated through a sensitivity analysis.

All analyses were performed in R version 4.4.1, using the *Mediation* (version 4.5.0) package.

### 2.7 Sensitivity Analyses

Three sensitivity analyses were conducted to evaluate the robustness of the findings. First, to address potential unmeasured mediator‐outcome confounding, a sensitivity analysis inspired by Ding and VanderWeele was performed [40]. This analysis quantified the degree of unmeasured confounding required to nullify the point estimate and the lower CIs of the NIE and NDE.

Second, to account for dependency between observations, a sensitivity analysis was performed restricting the sample to a random pregnancy per woman.

Third, to ensure the mediator, obstetric complications, preceded the outcome, PPD, women with no psychiatric history who were diagnosed with a psychiatric disorder (ICD‐10: F00‐99) or received psychotropic medication (ATC: N05‐06) during pregnancy for the first time were excluded.

### 2.8 Ethics

The study was approved by the Danish Data Protection Agency (through local registration at Aarhus University, journal number 2016‐051‐000001, serial number 2304). All data were anonymized and analyzed on a secure platform at Statistics Denmark. Informed consent was not required in accordance with Danish law.

## 3 Results

Of 170,218 mothers included in the study population, 40,707 (23.9%) had a personal psychiatric history (Table 1). Compared to their counterparts without a personal psychiatric history, they had a significantly higher risk of developing both PPD symptoms (13.4% vs. 6.1%) and PPD diagnosis (7.0% vs. 0.4%), had lower educational levels (mandatory: 19.1% vs. 6.6%), slightly higher parity (3+ children: 15.4% vs. 11.6%), were more often of Danish origin (91.6% vs. 84.9%), and experienced more complications (34.1% vs. 28.5%). See Table S1 for characteristics of women classified based on prescriptions alone, diagnosis alone, or both. For the analysis population, a washout period excluded 5028 women with a depression diagnosis or antidepressant prescription fills during pregnancy (Figure S3). The proportion of excluded women was mainly among women having a personal psychiatric history (11.7% vs. 0.2%). Additionally, complete case analysis was used, excluding women with missing data on covariates (*n* excluded = 1864), ending up with an analysis population of 163,326. The proportion of exclusions was comparable between the two groups (education: 0.2% vs. 0.4%, parity: 0.8% vs. 0.8%, preterm birth: 0.8% vs. 0.7%).

**TABLE 1:** Characteristics of the study population separated on personal psychiatric history.

Characteristics	Personal psychiatric history	
	No	Yes
*N* = 170,218	129,511 (76.1%)	40,707 (23.9%)
PPD symptoms		
No	121,641 (93.9%)	35,251 (86.6%)
Yes	7870 (6.1%)	5456 (13.4%)
PPD diagnosis		
No	128,987 (99.6%)	37,845 (93.0%)
Yes	524 (0.4%)	2862 (7.0%)
Age at delivery		
Mean (SD)	30.8 (4.7)	31.1 (5.0)
Education		
Mandatory	8590 (6.6%)	7764 (19.1%)
Short	36,596 (28.3%)	14,378 (35.3%)
Medium	7001 (5.4%)	1841 (4.5%)
High	76,788 (59.3%)	16,635 (40.9%)
Missing	536 (0.4%)	89 (0.2%)
Parity		
1 child	68,388 (52.8%)	20,100 (49.4%)
2 children	45,063 (34.8%)	13,996 (34.4%)
3+ children	15,081 (11.6%)	6280 (15.4%)
Missing	979 (0.8%)	331 (0.8%)
Calendar year of delivery		
2014^a^	2505 (1.9%)	744 (1.8%)
2015	16,172 (12.5%)	4877 (12.0%)
2016	18,415 (14.2%)	5790 (14.2%)
2017	19,613 (15.1%)	6205 (15.2%)
2018	20,867 (16.1%)	6431 (15.8%)
2019	20,924 (16.2%)	6576 (16.2%)
2020	15,863 (12.2%)	5174 (12.7%)
2021	15,152 (11.7%)	4910 (12.1%)
Origin of birth		
Denmark	109,929 (84.9%)	37,306 (91.6%)
Not Denmark	19,582 (15.1%)	3401 (8.4%)
Washout (ATC: N06A or ICD: F32‐33 during pregnancy)		
No	129,253 (99.8%)	35,937 (88.3%)
Yes	258 (0.2%)	4770 (11.7%)
Any complication		
No	91,773 (70.9%)	26,538 (65.2%)
Yes	36,870 (28.5%)	13,877 (34.1%)
Missing	868 (0.7%)	292 (0.7%)
Preeclampsia/eclampsia		
No	125,286 (96.7%)	39,048 (95.9%)
Yes	4225 (3.3%)	1659 (4.1%)
Gestational hypertension		
No	126,387 (97.6%)	39,530 (97.1%)
Yes	3124 (2.4%)	1177 (2.9%)
Gestational diabetes		
No	123,734 (95.5%)	38,160 (93.7%)
Yes	5777 (4.5%)	2547 (6.3%)
Hyperemesis gravidarum		
No	126,672 (97.8%)	39,065 (96.0%)
Yes	2839 (2.2%)	1642 (4.0%)
Postpartum hemorrhage		
No	119,807 (92.5%)	37,376 (91.8%)
Yes	9704 (7.5%)	3331 (8.2%)
C‐section		
No	111,519 (86.1%)	33,976 (83.5%)
Yes	17,992 (13.9%)	6731 (16.5%)
Preterm birth		
No	124,862 (96.4%)	38,901 (95.6%)
Yes	3724 (2.9%)	1491 (3.7%)
Missing	925 (0.7%)	315 (0.8%)

^a^ Some women gave birth in 2014; they are included because the EPDS screening was performed within 12 weeks postpartum, between January 1, 2015, and December 31, 2022.

**FIGURE 2:**
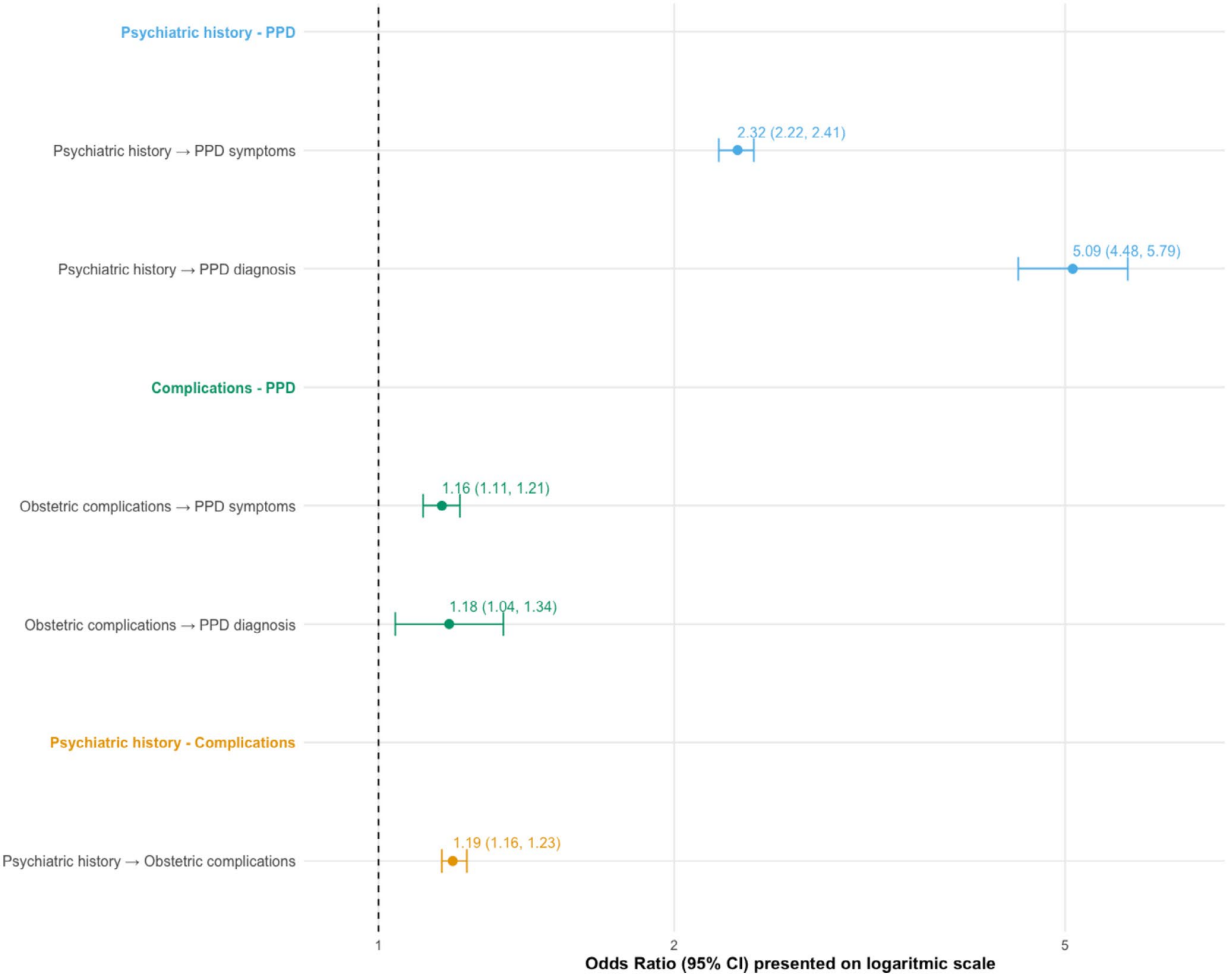
Logistic regressions estimating OR (95% CI) for the association separately between the exposure, mediator, and outcome. Association between psychiatric history and PPD was adjusted for parity, calendar year, educational level, and country of origin. Association between psychiatric history and complications was adjusted for parity, calendar year, maternal age, educational level, and country of origin. Association between complications and PPD was adjusted for personal psychiatric history, parity, calendar year, maternal age, educational level, and country of origin.

Figure 2 displays the associations between the exposure and outcome, the mediator and outcome, and the exposure and mediator (Step 1, Figure 1). Women with a psychiatric history had more than twofold higher odds (OR = 2.32 [95% CI, 2.22; 2.41]) of PPD symptoms and a more than fivefold higher odds (OR = 5.09 [95% CI, 4.48; 5.79]) of PPD diagnosis compared to those without a psychiatric history. Women with obstetric complications had an OR of 1.16 (95% CI, 1.11; 1.21) for PPD symptoms and an OR of 1.18 (95% CI, 1.04; 1.34) for PPD diagnosis compared to those without. Women with personal psychiatric history had an OR of 1.19 (95% CI, 1.16; 1.23) for obstetric complications. For specific complications, see Supporting Information (Table S2a,b).

Table S3 shows the association between the mediator and outcome, stratified by the exposure (Step 2, Figure 1). Most point estimates across strata fall within each other's 95% CI, suggesting limited evidence of effect modification by psychiatric history.

Table 2 displays the direct, indirect, and total mediating effects of obstetric complications in the association between psychiatric history and PPD, along with the proportion mediated (Step 3, Figure 1). See Supporting Information for specific complications (Table S4). Obstetric complications mediated 0.68% (0.50%; 1.00%) and 0.42% (0.14%; 0.79%) of the association between psychiatric history and PPD symptoms or diagnosis, respectively. The proportion mediated was similar for past episodes and recent episodes for PPD symptoms (0.62% [95% CI, 0.44%; 0.82%] vs. 0.73% [95% CI, 0.53%; 0.93%]) and slightly higher for past compared to recent episodes for PPD diagnosis (0.61% [95% CI, 0.14%; 1.09%] vs. 0.40% [95% CI, 0.04%; 0.81%]) (Table S5).

**TABLE 2:** Mediation analysis estimating OR (95% CI) and proportion mediated with personal psychiatric history as the exposure, complications as the mediator, and PPD as the outcome.

	Direct effect	Indirect effect	Total effect	Proportion mediated
PPD symptoms	1.07 (1.07; 1.07)	1.00 (1.00; 1.00)	1.07 (1.07; 1.07)	0.68% (0.50%; 1.00%)
PPD diagnosis	1.01 (1.01; 1.02)	1.00 (1.00; 1.00)	1.01 (1.01; 1.02)	0.42% (0.14%; 0.79%)

Sensitivity analyses (Tables S6 and S7) showed similar trends. The sensitivity analysis inspired by VanderWheele and Ding indicated that, in the absence of any observed mediational effect, only a minimal level of unmeasured confounding would be required to explain the observed association (Tables S8 and S9).

## 4 Discussion

For this study, we aimed to disentangle the complexity behind how major risk factors directly and indirectly influence PPD risk by applying mediation analysis to explore potential underlying mechanisms. Specifically, we sought to investigate whether the strongest PPD risk factor—personal psychiatric history—partly exerts its effect through other established risk factors, namely obstetric complications. These obstetric complications are themselves more prevalent among women with a psychiatric history, raising the question of whether they represent a pathway through which psychiatric vulnerability increases PPD risk. Using a stepwise approach, we first confirmed independent associations between psychiatric history, obstetric complications, and PPD. Second, the association between obstetric complications and PPD was similar in strata with and without psychiatric history. And third, given that psychiatric history is associated with adverse pregnancy outcomes, we hypothesized that obstetric complications would partially mediate the association between psychiatric history and PPD [17]. However, our findings did not support this hypothesis, as only 0.68% (95% CI, 0.50%; 1.00%, PPD symptoms) and 0.42% (95% CI, 0.14%; 0.79%, PPD diagnosis) of the observed association was explained by complications.

We confirmed that psychiatric history and obstetric complications were independently associated with PPD (Step 1, Figure 1). Our findings align with existing evidence, confirming the well‐established association between psychiatric history and both PPD symptoms and diagnosis, with a twofold and fivefold increase, respectively [5, 6, 7, 8, 9, 10, 11, 12, 13, 14, 15]. Compared to existing literature, our risk estimates of PPD diagnosis were somewhat higher than those reported on specific psychiatric disorders in an American study based on insurance claims. This divergence could possibly be due to population differences, as our study was population‐based within a universal healthcare system [12]. On the other hand, our risk estimate was much lower than in a Swedish population‐based study, which reported an almost twentyfold increase for PPD diagnosis. This may potentially be due to the washout period applied by us to ensure incident PPD cases [41]. Additionally, prior Danish research reported a low risk of PPD among mothers without a psychiatric history (0.6%), which we replicated (0.4%) [14]. Similarly, we confirmed that a wide range of obstetric complications were independently associated with PPD [9, 10, 18, 19, 20, 21, 22, 23, 24].

We showed that the association between obstetric complications and PPD was not modified by psychiatric history (Step 2, Figure 1), suggesting that obstetric complications increase the risk of PPD similarly among women with and without a psychiatric history. This contributes to a sparsely studied area, as most PPD research treats established risk factors as independent predictors, without examining whether they interact. As highlighted by Yim et al., interactive effects are rarely explored, and many studies include a broad set of predictors without explicitly examining how these factors may operate together or modify one another's effects [42]. Our results therefore add nuance to the evidence by providing results that two of the most well‐established PPD risk factors appear to operate independently, rather than synergistically.

We did not find that obstetric complications mediate the association between psychiatric history and PPD (Step 3, Figure 1). Despite a recent study establishing a clear link between maternal psychiatric history and adverse neonatal outcomes, our hypothesis that the association between psychiatric history and PPD would be partially mediated through obstetric complications was not supported [17]. This suggests that while obstetric complications contribute to PPD risk, they do not explain the pathway through which psychiatric history influences PPD development.

PPD affects a substantial number of mothers, yet it remains challenging to predict who will develop the disorder. We aimed to enhance the understanding of mechanisms underlying the disorder, as it may inform targeted strategies, ideally aiming to prevent PPD from developing. Our study contributes to this etiological understanding by examining whether obstetric complications mediate the association between psychiatric history and PPD. The finding that obstetric complications do not explain any part of this association suggests that these risk factors operate through distinct pathways. Although psychiatric history is not a modifiable risk factor per se and thus cannot be targeted directly to prevent PPD, it remains a critical predictor. Gaining deeper insights into how psychiatric vulnerability contributes to PPD development is essential for improving early identification and design of supportive interventions tailored to women at risk. Our findings highlight the need for further research into alternative mechanisms, such as biological, psychological, or social factors, to refine our understanding of PPD etiology and onset.

Broadly, our findings suggest that both women with a psychiatric history and those with obstetric complications should receive close attention to enhance early identification and support. However, as our results indicate that these risk factors operate through separate pathways, clinical practice should consider tailored approaches. Women with a psychiatric history may benefit from early psychological support and monitoring throughout pregnancy and the postpartum. While those experiencing complications might require targeted interventions addressing the specific physical and emotional stressors associated with adverse obstetric outcomes, such as pain management, support during recovery, and reassurance around offspring health. Developing differentiated care pathways that reflect the distinct needs and mechanisms underlying each risk factor could improve intervention efforts. This approach also aligns with a more personalized model of perinatal mental healthcare, emphasizing risk stratification and timely, appropriate support based on individual risk profiles.

### 4.1 Strengths and Limitations

A key strength of this study is the use of the HOPE cohort, a population‐based cohort representative of the Danish population. By linking HOPE with population‐wide registers, this ensures a comprehensive coverage of the Danish source population on a wide range of information [26]. Additionally, HOPE provides a broad spectrum of PPD cases, including cases defined from EPDS scores, antidepressant use, and hospital diagnoses.

We followed the AGReMA guideline to ensure transparency and robustness in the conduct and reporting of our mediation analysis. However, as recently highlighted, specific challenges frequently arise in psychiatric mediation studies [43]. Namely, lack of clear temporal ordering, insufficient control for confounding, and failure to consider exposure‐mediator interaction. We addressed these concerns in several ways. First, we leveraged longitudinal data to establish temporal ordering and further assessed the robustness of this ordering in a sensitivity analysis excluding mothers who received their first‐ever psychiatric diagnosis between conception and delivery. Second, we constructed an a priori DAG to clarify the assumed causal structure and guide confounder adjustment. Finally, we assessed interaction between exposure and mediator to avoid misspecification of the underlying model.

Despite these strengths, our study also has limitations. First, the HOPE cohort is underrepresented in non‐Danish populations, limiting generalizability to immigrant populations [26]. Second, although we adjusted for key sociodemographic and reproductive factors (education, parity, maternal age at delivery, origin of birth, and calendar year of delivery), some potential confounders were not available in the registers, including physical activity [44], social support [45, 46], and unintended pregnancy [47]. Such unmeasured confounding could affect estimates of both direct and indirect effects and should be taken into account when interpreting the results of the mediation analysis (Figure S2). In addition, while our study focused on obstetric complications as mediators, we acknowledge that adverse neonatal outcomes could plausibly represent an additional pathway linking obstetric complications and PPD [17]. As adverse neonatal outcomes were not included in the mediation model, some indirect pathways may not be fully captured. Future studies could investigate this further. Third, while we adjusted for maternal education as a potential confounder, it is possible that early‐life psychiatric history influenced educational attainment for some women. Consequently, education may represent a pathway between exposure and outcome for certain individuals, rather than a pure confounder. Thus, adjusting for education may have resulted in some overadjustment, potentially attenuating the observed association. Finally, we dichotomized the EPDS score to define PPD symptoms using a cut‐off ≥ 11. While this approach identifies clinically relevant cases, it may result in some loss of information on the full range of symptom severity and precludes assessment of dose–response relationships between obstetric complications and depressive symptoms. However, separate analyses of individual obstetric complications were conducted (see Supporting Information), providing some insights into the differential associations of specific complications with PPD risk.

## 5 Conclusion

In this population‐based cohort study, we first confirmed that personal psychiatric history and obstetric complications independently were associated with PPD. Second, we found no evidence of interaction between psychiatric history and obstetric complications. And thirdly, obstetric complications did not mediate the association between personal psychiatric history and PPD, suggesting that these risk factors operate through distinct pathways. These findings highlight the importance of tailored approaches: women with a psychiatric history may benefit from proactive, continuous mental health care during the perinatal period, whereas those experiencing obstetric complications may require targeted screening and support in the immediate postpartum phase. Our results underscore the need for differentiated strategies that reflect the specific needs and risk profiles of distinct high‐risk groups, and they call for further research into the mechanisms linking psychiatric vulnerability to PPD to better inform such precision intervention efforts.

## Author Contributions

Mette‐Marie Zacher Kjeldsen and Katrine Holde had full access to all the data in the study and takes responsibility for the integrity of the data and the accuracy of the data analysis. Concept and design: All authors. Acquisition, analysis, or interpretation of data: Mette‐Marie Zacher Kjeldsen, Katrine Holde, Liselotte Vogdrup Petersen, and Trine Munk‐Olsen. Drafting of the manuscript: Mette‐Marie Zacher Kjeldsen. Critical revision of the manuscript for important intellectual content: All authors. Statistical analysis: Katrine Holde. Obtained funding: Liselotte Vogdrup Petersen and Trine Munk‐Olsen. Administrative, technical, or material support: N/A. Supervision: Liselotte Vogdrup Petersen and Trine Munk‐Olsen.

## Funding

M.M.Z.‐K. is supported by the Lundbeck Foundation (grant nos. 32691 and R433‐2023‐565). T.M.‐O. and M.L.M. are also supported by the Lundbeck Foundation (grant no. 32691). V.B., E.E.B., and T.M.‐O. are funded by the National Institute of Mental Health (NIMH, grant no. R01MH122869). The funding agencies had no role in the design, data collection, analysis, interpretation, writing the manuscript, or the decision to submit the manuscript for publication.

## Disclosure

The corresponding author (M.‐M.Z.K.) confirms that the manuscript represents an honest, accurate, and transparent account of the study's finding.

## Conflicts of Interest

K.B.M. has received a speaker's fee from Medice Nordic within the last 3 years. T.M.‐O. has received a speaker's fee from Lundbeck A/S within the last 3 years. The other authors declare no conflicts of interest.

## Supporting information


**Figure S1:** Illustration of population, exposure, mediator, and outcome in relation to temporal order.
**Figure S2:** Directed acyclic graph (DAG) on the confounding structure.
**Table S1:** Characteristics of study population separated on prescriptions and diagnosis for personal psychiatric history.
**Figure S3:** Flowchart from the study population to the analysis population.
**Table S2:** (a) Logistic regressions estimating OR (95% CI) for the association between the exposure and outcome, as well as the mediator and outcome. (b) Logistic regressions estimating OR (95% CI) for the association between the exposure and mediator.
**Table S3:** Logistic regressions estimating OR (95% CI) for the mediator stratified by the exposure.
**Table S4:** Mediation analysis estimating OR (95% CI) and the proportion mediated for specific complications.
**Table S5:** Mediation analysis estimating OR (95% CI) and the proportion mediated stratified by timing of the exposure (recent and past).
**Table S6:** Sensitivity analysis of mediation analysis restricted to one random birth per mother in HOPE.
**Table S7:** Sensitivity analysis of mediation analysis with exclusion of women with no prior psychiatric history who receive a psychiatric diagnosis or psychotropic medication from conception until delivery.
**Table S8:** Sensitivity analysis estimating the *E* value for the indirect effect.
**Table S9:** Sensitivity analysis estimating the *E* value for the direct effect.

## Data Availability

Data used in this study are derived from healthcare records and Danish national registers and contain sensitive personal information. In accordance with Danish data protection regulations, individual‐level data cannot be shared publicly. Access may be granted to researchers who meet the criteria for access to confidential data through application to Statistics Denmark.

## References

[1] J. Hahn‐Holbrook , T. Cornwell‐Hinrichs , and I. Anaya , “Economic and Health Predictors of National Postpartum Depression Prevalence: A Systematic Review, Meta‐Analysis, and Meta‐Regression of 291 Studies From 56 Countries,” Frontiers in Psychiatry 8 (2018): 248, 10.3389/fpsyt.2017.00248. 29449816 PMC5799244

[2] Z. Wang , J. Liu , H. Shuai , et al., “Mapping Global Prevalence of Depression Among Postpartum Women,” Translational Psychiatry 11, no. 1 (2021): 543, 10.1038/s41398-021-01663-6. 34671011 PMC8528847

[3] A. Fonseca , A. Ganho‐Ávila , M. Lambregtse‐van Den Berg , et al., “Emerging Issues and Questions on Peripartum Depression Prevention, Diagnosis and Treatment: A Consensus Report From the Cost Action Riseup‐PPD,” Journal of Affective Disorders 274 (2020): 167–173, 10.1016/j.jad.2020.05.112. 32469800

[4] K. Gidén , L. Vinnerljung , S. I. Iliadis , E. Fransson , and A. Skalkidou , “Feeling Better? – Identification, Interventions, and Remission Among Women With Early Postpartum Depressive Symptoms in Sweden: A Nested Cohort Study,” European Psychiatry 67, no. 1 (2024): e14, 10.1192/j.eurpsy.2024.6. 38254262 PMC10897831

[5] B. F. Hutchens and J. Kearney , “Risk Factors for Postpartum Depression: An Umbrella Review,” Journal of Midwifery & Women's Health 65, no. 1 (2020): 96–108, 10.1111/jmwh.13067. 31970924

[6] J. Guintivano , T. Manuck , and S. Meltzer‐Brody , “Predictors of Postpartum Depression: A Comprehensive Review of the Last Decade of Evidence,” Clinical Obstetrics and Gynecology 61, no. 3 (2018): 591–603, 10.1097/GRF.0000000000000368. 29596076 PMC6059965

[7] L. M. Howard , E. Molyneaux , C. L. Dennis , T. Rochat , A. Stein , and J. Milgrom , “Non‐Psychotic Mental Disorders in the Perinatal Period,” Lancet 384, no. 9956 (2014): 1775–1788, 10.1016/S0140-6736(14)61276-9. 25455248

[8] X. H. Zhao and Z. H. Zhang , “Risk Factors for Postpartum Depression: An Evidence‐Based Systematic Review of Systematic Reviews and Meta‐Analyses,” Asian Journal of Psychiatry 53 (2020): 102353, 10.1016/j.ajp.2020.102353. 32927309

[9] X. Liu , S. Wang , and G. Wang , “Prevalence and Risk Factors of Postpartum Depression in Women: A Systematic Review and Meta‐Analysis,” Journal of Clinical Nursing 31, no. 19–20 (2022): 2665–2677, 10.1111/jocn.16121. 34750904

[10] T. Munk‐Olsen , X. Liu , K. B. Madsen , et al., “Postpartum Depression: A Developed and Validated Model Predicting Individual Risk in New Mothers,” Translational Psychiatry 12, no. 1 (2022): 419, 10.1038/s41398-022-02190-8. 36180471 PMC9525696

[11] Y. Zhang , S. Wang , A. Hermann , R. Joly , and J. Pathak , “Development and Validation of a Machine Learning Algorithm for Predicting the Risk of Postpartum Depression Among Pregnant Women,” Journal of Affective Disorders 279 (2021): 1–8, 10.1016/j.jad.2020.09.113. 33035748 PMC7738412

[12] S. L. Johansen , B. A. Stenhaug , T. K. Robakis , K. E. Williams , and M. R. Cullen , “Past Psychiatric Conditions as Risk Factors for Postpartum Depression: A Nationwide Cohort Study,” Journal of Clinical Psychiatry 81, no. 1 (2020): m12929, 10.4088/JCP.19m12929. 31967747

[13] A. Andersson , M. Garcia‐Argibay , A. Viktorin , et al., “Depression and Anxiety Disorders During the Postpartum Period in Women Diagnosed With Attention Deficit Hyperactivity Disorder,” Journal of Affective Disorders 325 (2023): 817–823, 10.1016/j.jad.2023.01.069. 36681302

[14] M. L. H. Rasmussen , M. Strøm , J. Wohlfahrt , P. Videbech , and M. Melbye , “Risk, Treatment Duration, and Recurrence Risk of Postpartum Affective Disorder in Women With no Prior Psychiatric History: A Population‐Based Cohort Study,” PLoS Medicine 14, no. 9 (2017): e1002392, 10.1371/journal.pmed.1002392. 28949960 PMC5614423

[15] T. Munk‐Olsen , K. G. Ingstrup , B. M. Johannsen , and X. Liu , “Population‐Based Assessment of the Recurrence Risk of Postpartum Mental Disorders: Will It Happen Again?,” JAMA Psychiatry 77, no. 2 (2020): 213–214, 10.1001/jamapsychiatry.2019.3208. 31617870 PMC6802053

[16] M. Rusner , M. Berg , and C. Begley , “Bipolar Disorder in Pregnancy and Childbirth: A Systematic Review of Outcomes,” BMC Pregnancy and Childbirth 16, no. 1 (2016): 331, 10.1186/s12884-016-1127-1. 27793111 PMC5084442

[17] N. C. Momen , H. Chatwin , K. Holde , et al., “Maternal Mental Disorders and Neonatal Outcomes: Danish Population‐Based Cohort Study,” British Journal of Psychiatry 8 (2024): 1–8, 10.1192/bjp.2024.164. PMC11781859 39376122

[18] L. Caropreso , C. T. de Azevedo , M. Eltayebani , and B. N. Frey , “Preeclampsia as a Risk Factor for Postpartum Depression and Psychosis: A Systematic Review and Meta‐Analysis,” Archives of Women's Mental Health 23, no. 4 (2020): 493–505, 10.1007/s00737-019-01010-1. 31802249

[19] A. Sweeting , W. Hannah , H. Backman , et al., “Epidemiology and Management of Gestational Diabetes,” Lancet 404, no. 10448 (2024): 175–192, 10.1016/S0140-6736(24)00825-0. 38909620

[20] S. Meltzer‐Brody , M. L. Maegbaek , S. E. Medland , W. C. Miller , P. Sullivan , and T. Munk‐Olsen , “Obstetrical, Pregnancy and Socio‐Economic Predictors for New‐Onset Severe Postpartum Psychiatric Disorders in Primiparous Women,” Psychological Medicine 47, no. 8 (2017): 1427–1441, 10.1017/S0033291716003020. 28112056 PMC5429203

[21] G. Schoretsanitis , C. Gastaldon , N. Ochsenbein‐Koelble , S. Olbrich , C. Barbui , and E. Seifritz , “Postpartum Hemorrhage and Postpartum Depression: A Systematic Review and Meta‐Analysis of Observational Studies,” Acta Psychiatrica Scandinavica 150, no. 5 (2024): 274–283, 10.1111/acps.13583. 37286177

[22] L. Sun , S. Wang , and X. Q. Li , “Association Between Mode of Delivery and Postpartum Depression: A Systematic Review and Network Meta‐Analysis,” Australian and New Zealand Journal of Psychiatry 55, no. 6 (2021): 588–601, 10.1177/0004867420954284. 32929976

[23] J. A. F. de Paula Eduardo , M. G. de Rezende , P. R. Menezes , and C. M. Del‐Ben , “Preterm Birth as a Risk Factor for Postpartum Depression: A Systematic Review and Meta‐Analysis,” Journal of Affective Disorders 259 (2019): 392–403, 10.1016/j.jad.2019.08.069. 31470184

[24] R. L. Goldenberg , J. F. Culhane , J. D. Iams , and R. Romero , “Epidemiology and Causes of Preterm Birth,” Lancet 371, no. 9606 (2008): 75–84, 10.1016/S0140-6736(08)60074-4. 18177778 PMC7134569

[25] H. Lee , A. G. Cashin , S. E. Lamb , et al., “A Guideline for Reporting Mediation Analyses of Randomized Trials and Observational Studies: The AGReMA Statement,” Journal of the American Medical Association 326, no. 11 (2021): 1045–1056, 10.1001/jama.2021.14075. 34546296 PMC8974292

[26] M. M. Zacher Kjeldsen , M. L. Mægbæk , X. Liu , et al., “The HOPE Cohort: Cohort Profile and Evaluation of Selection Bias,” European Journal of Epidemiology 39, no. 8 (2024): 943–954, 10.1007/s10654-024-01150-4. 39158818 PMC11410971

[27] J. L. Cox , J. M. Holden , R. Sagovsky , and Detection of Postnatal Depression , “Development of the 10‐Item Edinburgh Postnatal Depression Scale,” British Journal of Psychiatry 150 (1987): 782–786, 10.1192/bjp.150.6.782. 3651732

[28] O. Mors , G. P. Perto , and P. B. Mortensen , “The Danish Psychiatric Central Research Register,” Scandinavian Journal of Public Health 39, no. 7 (2011): 54–57, 10.1177/1403494810395825. 21775352

[29] A. Pottegård , S. A. J. Schmidt , H. Wallach‐Kildemoes , H. T. Sørensen , J. Hallas , and M. Schmidt , “Data Resource Profile: The Danish National Prescription Registry,” International Journal of Epidemiology 46, no. 3 (2017): 798, 10.1093/ije/dyw213. 27789670 PMC5837522

[30] M. Schmidt , S. A. J. Schmidt , J. L. Sandegaard , V. Ehrenstein , L. Pedersen , and H. T. Sørensen , “The Danish National Patient Registry: A Review of Content, Data Quality, and Research Potential,” Clinical Epidemiology 7 (2015): 449–490, 10.2147/CLEP.S91125. 26604824 PMC4655913

[31] J. Smith‐Nielsen , S. Matthey , T. Lange , and M. S. Væver , “Validation of the Edinburgh Postnatal Depression Scale Against Both DSM‐5 and ICD‐10 Diagnostic Criteria for Depression,” BMC Psychiatry 18, no. 1 (2018): 393, 10.1186/s12888-018-1965-7. 30572867 PMC6302501

[32] A. Borovac‐Pinheiro , R. C. Pacagnella , J. G. Cecatti , et al., “Postpartum Hemorrhage: New Insights for Definition and Diagnosis,” American Journal of Obstetrics and Gynecology 219, no. 2 (2018): 162–168, 10.1016/j.ajog.2018.04.013. 29660298

[33] M. Bliddal , A. Broe , A. Pottegård , J. Olsen , and J. Langhoff‐Roos , “The Danish Medical Birth Register,” European Journal of Epidemiology 33, no. 1 (2018): 27–36, 10.1007/s10654-018-0356-1. 29349587

[34] C. B. Pedersen , “The Danish Civil Registration System,” Scandinavian Journal of Public Health 39, no. 7 (2011): 22–25, 10.1177/1403494810387965. 21775345

[35] V. M. Jensen and A. W. Rasmussen , “Danish Education Registers,” Scandinavian Journal of Public Health 39, no. 7 (2011): 91–94, 10.1177/1403494810394715. 21775362

[36] UNESCO , International Standard Classification of Education: ISCED 2011 (UNESCO Institute for Statistics, 2012).

[37] J. J. M. Rijnhart , M. J. Valente , H. L. Smyth , and D. P. MacKinnon , “Statistical Mediation Analysis for Models With a Binary Mediator and a Binary Outcome: The Differences Between Causal and Traditional Mediation Analysis,” Prevention Science 24, no. 3 (2023): 408–418, 10.1007/s11121-021-01308-6. 34782926 PMC9108123

[38] T. J. VanderWeele and S. Vansteelandt , “Odds Ratios for Mediation Analysis for a Dichotomous Outcome,” American Journal of Epidemiology 172, no. 12 (2010): 1339–1348, 10.1093/aje/kwq332. 21036955 PMC2998205

[39] J. J. M. Rijnhart , S. J. Lamp , M. J. Valente , D. P. MacKinnon , J. W. R. Twisk , and M. W. Heymans , “Mediation Analysis Methods Used in Observational Research: A Scoping Review and Recommendations,” BMC Medical Research Methodology 21, no. 1 (2021): 226, 10.1186/s12874-021-01426-3. 34689754 PMC8543973

[40] P. Ding and T. J. Vanderweele , “Sharp Sensitivity Bounds for Mediation Under Unmeasured Mediator‐Outcome Confounding,” Biometrika 103, no. 2 (2016): 483–490, 10.1093/biomet/asw012. 27279672 PMC4890130

[41] M. E. Silverman , A. Reichenberg , D. A. Savitz , et al., “The Risk Factors for Postpartum Depression: A Population‐Based Study,” Depression and Anxiety 34, no. 2 (2017): 178–187, 10.1002/da.22597. 28098957 PMC5462547

[42] I. S. Yim , L. R. Tanner Stapleton , C. M. Guardino , J. Hahn‐Holbrook , and C. D. Schetter , “Biological and Psychosocial Predictors of Postpartum Depression: Systematic Review and Call for Integration,” Annual Review of Clinical Psychology 11 (2015): 99–137, 10.1146/annurev-clinpsy-101414-020426. PMC5659274 25822344

[43] Z. C. Narita , M. Miyashita , T. A. Furukawa , and A. Nishida , “Key Considerations in Mediation Analysis for Psychiatric Research,” JAMA Psychiatry 82 (2025): 634–636, 10.1001/jamapsychiatry.2025.0566. 40266608

[44] M. Rahmati , S. Lee , D. K. Yon , et al., “Physical Activity and Prevention of Mental Health Complications: An Umbrella Review,” Neuroscience and Biobehavioral Reviews 160 (2024): 105641, 10.1016/j.neubiorev.2024.105641. 38527637

[45] A. Bedaso , J. Adams , W. Peng , and D. Sibbritt , “The Relationship Between Social Support and Mental Health Problems During Pregnancy: A Systematic Review and Meta‐Analysis,” Reproductive Health 18, no. 1 (2021): 162, 10.1186/s12978-021-01209-5. 34321040 PMC8320195

[46] M. M. E. Riem , M. J. Bakermans‐Kranenburg , M. Cima , and M. H. van IJzendoorn , “Grandparental Support and Maternal Postpartum Mental Health: A Review and Meta‐Analysis,” Human Nature 34, no. 1 (2023): 25–45, 10.1007/s12110-023-09440-8. 36750511 PMC9905757

[47] C. Gastaldon , M. Solmi , C. U. Correll , C. Barbui , and G. Schoretsanitis , “Risk Factors of Postpartum Depression and Depressive Symptoms: Umbrella Review of Current Evidence From Systematic Reviews and Meta‐Analyses of Observational Studies,” British Journal of Psychiatry 221, no. 4 (2022): 591–602, 10.1192/bjp.2021.222. 35081993

