# Supplementary material for: Psychiatric History and Postpartum Depression: The Mediating Role of Obstetric Complications
Source: Acta Psychiatr Scand. 2026 May 5;154(3):223–33. doi: 10.1111/acps.70105 (PMC13429344; doi:10.1111/acps.70105)
Supplement: Supplementary file 1 — Figure S1: Illustration of population, exposure, mediator, and outcome in relation to temporal order. Figure S2: Directed acyclic graph (DAG) on the confounding structure. Table S1: Characteristics of study population separated on prescriptions and diagnosis for personal psychiatric history. Figure S3: Flowchart from the study population to the analysis population. Table S2: (a) Logistic regressions estimating OR (95% CI) for the association between the exposure and outcome, as well as the mediator and outcome. (b) Logistic regressions estimating OR (95% CI) for the association between the exposure and mediator. Table S3: Logistic regressions estimating OR (95% CI) for the mediator stratified by the exposure. Table S4: Mediation analysis estimating OR (95% CI) and the proportion mediated for specific complications. Table S5: Mediation analysis estimating OR (95% CI) and the proportion mediated stratified by timing of the exposure (recent and past). Table S6: Sensitivity analysis of mediation analysis restricted to one random birth per mother in HOPE. Table S7: Sensitivity analysis of mediation analysis with exclusion of women with no prior psychiatric history who receive a psychiatric diagnosis or psychotropic medication from conception until delivery. Table S8: Sensitivity analysis estimating the E value for the indirect effect. Table S9: Sensitivity analysis estimating the E value for the direct effect. [file ACPS-154-223-s001.pdf]

Supplementary Online Content

eFigure 1 Illustration of population, exposure, mediator, and outcome in relation to temporal order

eFigure 2 Directed Acyclic Graph (DAG) on the confounding structure

eTable 1 Characteristics of study population separated on prescriptions and diagnosis for personal psychiatric history

eFigure 3 Flowchart from the study population to the analysis population

eTable 2a Logistic regressions estimating OR (95% CI) for the association between the exposure and outcome, as well as the mediator and outcome

eTable 2b Logistic regressions estimating OR (95% CI) for the association between the exposure and mediator

eTable 3 Logistic regressions estimating OR (95% CI) for the mediator stratified by the exposure

eTable 4 Mediation analysis estimating OR (95% CI) and the proportion mediated for specific complications

eTable 5 Mediation analysis estimating OR (95% CI) and the proportion mediated stratified by timing of the exposure (recent and past)

eTable 6 Sensitivity analysis of mediation analysis restricted to one random birth per mother in HOPE

eTable 7 Sensitivity analysis of mediation analysis with exclusion of women with no prior psychiatric history who receive a psychiatric diagnosis or psychotropic medication from conception until delivery

eTable 8 Sensitivity analysis estimating the E-value for the indirect effect

eTable 9 Sensitivity analysis estimating the E-value for the direct effect

Note:
- Exposure = Personal psychiatric history
- Mediator = Obstetric complications
- Outcome = PPD

eFigure 1. Illustration of population, exposure, mediator, and outcome in relation to temporal order

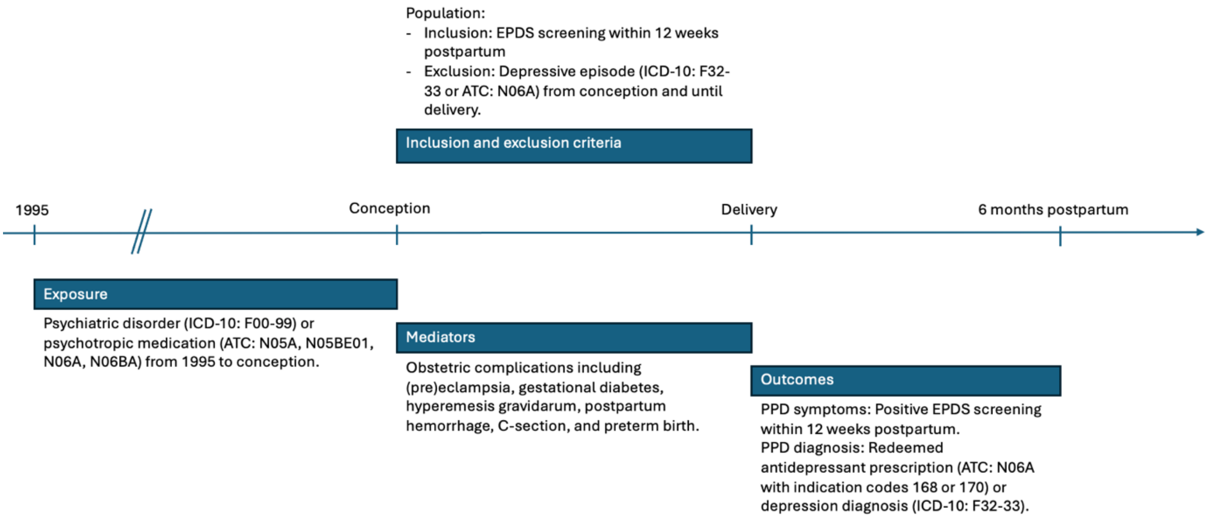

eFigure 2. Directed Acyclic Graph (DAG) on the confounding structure

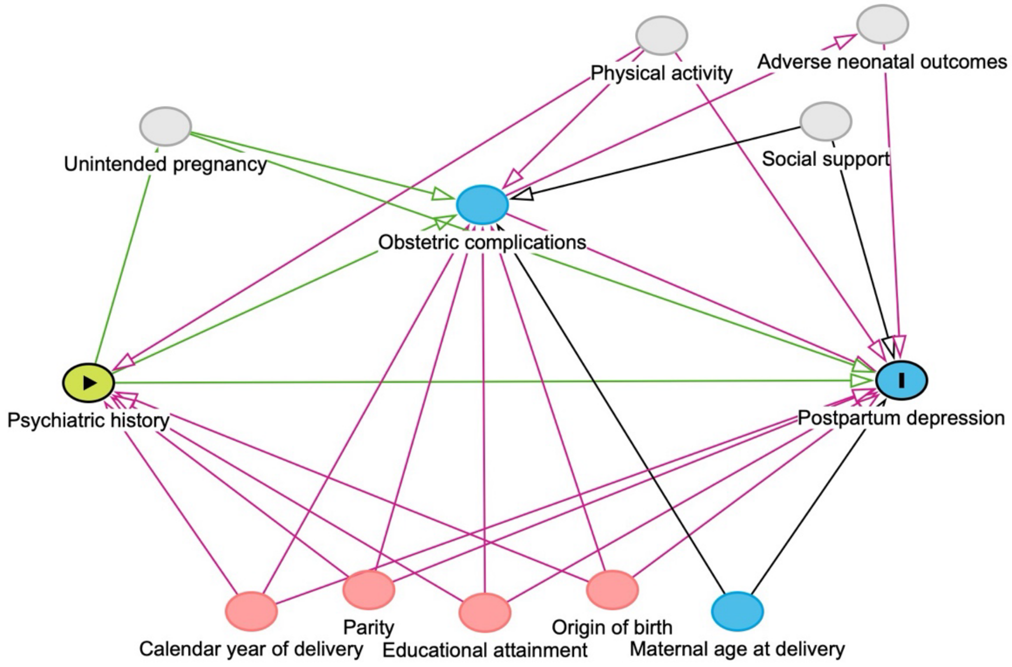

eTable 1. Characteristics of study population separated on prescriptions and diagnosis for personal psychiatric history

Characteristic	Personal psychiatric history<br>None<br>N = 129,511	Prescription only<br>N = 10,351	Diagnosis only<br>N = 2,890	Diagnosis and prescription<br>N = 27,466
**PPD**				
No	121,641 (93.9%)	8,801 (85.0%)	2,438 (84.4%)	24,012 (87.4%)
Yes	7,870 (6.1%)	1,550 (15.0%)	452 (15.6%)	3,454 (12.6%)
**Severe PPD**				
No	128,987 (99.6%)	8,622 (83.3%)	2,824 (97.7%)	26,399 (96.1%)
Yes	524 (0.4%)	1,729 (16.7%)	66 (2.3%)	1,067 (3.9%)
**Age at delivery**				
Mean (SD)	30.8 (4.7)	31.2 (5.0)	28.6 (5.2)	31.3 (5.0)
**Education**				
Mandatory	8,590 (6.6%)	1,978 (19.1%)	944 (32.7%)	4,842 (17.6%)
Short	36,596 (28.3%)	3,777 (36.5%)	936 (32.4%)	9,665 (35.2%)
Medium	7,001 (5.4%)	450 (4.3%)	117 (4.0%)	1,274 (4.6%)
High	76,788 (59.3%)	4,117 (39.8%)	883 (30.6%)	11,635 (42.4%)
Missing	536 (0.4%)	29 (0.3%)	10 (0.3%)	50 (0.2%)
**Parity**				
1	68,388 (52.8%)	5,506 (53.2%)	1,696 (58.7%)	12,898 (47.0%)
2	45,063 (34.8%)	3,119 (30.1%)	872 (30.2%)	10,005 (36.4%)
3+	15,081 (11.6%)	1,657 (16.0%)	310 (10.7%)	4,313 (15.7%)
Missing	979 (0.8%)	69 (0.7%)	12 (0.4%)	250 (0.9%)
**Calendar year of delivery**				
2014*	2,505 (1.9%)	260 (2.5%)	40 (1.4%)	444 (1.6%)
2015	16,172 (12.5%)	1,569 (15.2%)	292 (10.1%)	3,016 (11.0%)
2016	18,415 (14.2%)	1,671 (16.1%)	380 (13.1%)	3,739 (13.6%)
2017	19,613 (15.1%)	1,586 (15.3%)	434 (15.0%)	4,185 (15.2%)
2018	20,867 (16.1%)	1,486 (14.4%)	473 (16.4%)	4,472 (16.3%)
2019	20,924 (16.2%)	1,524 (14.7%)	490 (17.0%)	4,562 (16.6%)
2020	15,863 (12.2%)	1,154 (11.1%)	414 (14.3%)	3,606 (13.1%)
2021	15,152 (11.7%)	1,101 (10.6%)	367 (12.7%)	3,442 (12.5%)
**Origin of birth**				
Denmark	109,929 (84.9%)	9,334 (90.2%)	2,582 (89.3%)	25,390 (92.4%)
Not Denmark	19,582 (15.1%)	1,017 (9.8%)	308 (10.7%)	2,076 (7.6%)
**Washout**				
No	129,253 (99.8%)	7,221 (69.8%)	2,829 (97.9%)	25,887 (94.3%)
Yes	258 (0.2%)	3,130 (30.2%)	61 (2.1%)	1,579 (5.7%)
**Any complication**				
No	91,773 (70.9%)	6,500 (62.8%)	1,969 (68.1%)	18,069 (65.8%)
Yes	36,870 (28.5%)	3,792 (36.6%)	911 (31.5%)	9,174 (33.4%)
Missing	868 (0.7%)	59 (0.6%)	10 (0.3%)	223 (0.8%)
**Preeclampsia/eclampsia**				
No	125,286 (96.7%)	9,873 (95.4%)	2,782 (96.3%)	26,393 (96.1%)
Yes	4,225 (3.3%)	478 (4.6%)	108 (3.7%)	1,073 (3.9%)
**Gestational hypertension**				
No	126,387 (97.6%)	10,037 (97.0%)	2,824 (97.7%)	26,669 (97.1%)
Yes	3,124 (2.4%)	314 (3.0%)	66 (2.3%)	797 (2.9%)
**Gestational diabetes**				
No	123,734 (95.5%)	9,650 (93.2%)	2,726 (94.3%)	25,784 (93.9%)
Yes	5,777 (4.5%)	701 (6.8%)	164 (5.7%)	1,682 (6.1%)
**Hyperemesis gravidarum**				
No	126,672 (97.8%)	9,833 (95.0%)	2,774 (96.0%)	26,458 (96.3%)
Yes	2,839 (2.2%)	518 (5.0%)	116 (4.0%)	1,008 (3.7%)
**Postpartum hemorrhage**				
No	119,807 (92.5%)	9,482 (91.6%)	2,685 (92.9%)	25,209 (91.8%)
Yes	9,704 (7.5%)	869 (8.4%)	205 (7.1%)	2,257 (8.2%)
**C-section**				

	Personal psychiatric history			
**Characteristic**	**None N = 129,511**	**Prescription only N = 10,351**	**Diagnosis only N = 2,890**	**Diagnosis and prescription N = 27,466**
No	111,519 (86.1%)	8,560 (82.7%)	2,431 (84.1%)	22,985 (83.7%)
Yes	17,992 (13.9%)	1,791 (17.3%)	459 (15.9%)	4,481 (16.3%)
**Preterm birth**				
No	124,862 (96.4%)	9,862 (95.3%)	2,775 (96.0%)	26,264 (95.6%)
Yes	3,724 (2.9%)	424 (4.1%)	104 (3.6%)	963 (3.5%)
Missing	925 (0.7%)	65 (0.6%)	11 (0.4%)	239 (0.9%)

* Some women gave birth in 2014; they are included because the EPDS screening was performed within 12 weeks postpartum, between January 1, 2015, and December 31, 2022.

eFigure 3. Flowchart from the study population to the analysis population

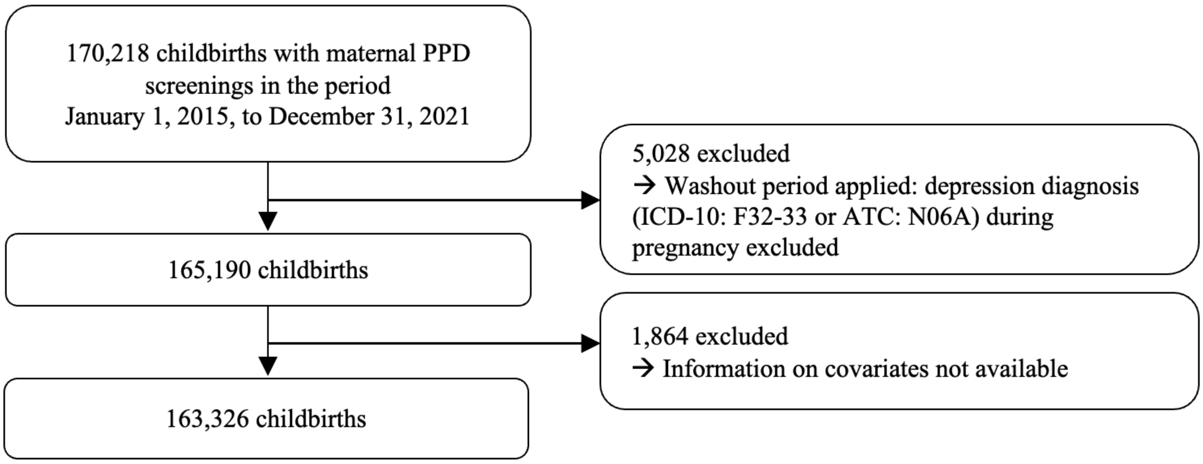

eTable 2a. Logistic regressions estimating OR (95% CI) for the association between the exposure and outcome, as well as the mediator and outcome

	PPD symptoms		PPD diagnosis	
	Exposure*	Mediator*	Exposure*	Mediator*
Preeclampsia/eclampsia	2.32 (2.22;2.41)	1.16 (1.05;1.27)	5.09 (4.48;5.79)	1.02 (0.74;1.41)
Gestational hypertension	2.32 (2.22;2.41)	1.03 (0.92;1.16)	5.09 (4.48;5.79)	0.87 (0.58;1.30)
Gestational diabetes	2.32 (2.22;2.41)	1.12 (1.03;1.21)	5.09 (4.48;5.79)	1.14 (0.89;1.46)
Hyperemesis gravidarum	2.32 (2.22;2.41)	1.39 (1.25;1.54)	5.09 (4.48;5.79)	1.70 (1.30;2.23)
Postpartum hemorrhage	2.32 (2.22;2.41)	1.05 (0.98;1.12)	5.09 (4.48;5.79)	1.05 (0.84;1.31)
Acute C-section	2.32 (2.22;2.41)	1.16 (1.10;1.22)	5.09 (4.48;5.79)	1.19 (1.02;1.40)
Preterm birth	2.32 (2.22;2.41)	1.34 (1.21;1.47)	5.09 (4.48;5.79)	1.25 (0.92;1.70)

* Association between psychiatric history and PPD were adjusted for parity, calendar year, educational level, and country of origin. Association between complications and PPD were adjusted for personal psychiatric history, parity, calendar year, maternal age, educational level, and country of origin.

E.g., for Preeclampsia/eclampsia the column with "Exposure" (psychiatric history) expresses increased OR of PPD among women with psychiatric history adjusted for preeclampsia/eclampsia. The column with "Mediator" expresses increased OR of PPD among women with preeclampsia/eclampsia adjusted for psychiatric history.

eTable 2b. Logistic regression estimating OR (95% CI) for the association between the exposure and mediator

Associations*	OR (95% CI)
Personal psychiatric history - Preeclampsia/eclampsia	1.13 (1.06;1.20)
Personal psychiatric history - Gestational hypertension	1.12 (1.03;1.20)
Personal psychiatric history - Gestational diabetes	1.23 (1.16;1.30)
Personal psychiatric history - Hyperemesis gravidarum	1.72 (1.60;1.84)
Personal psychiatric history - Postpartum hemorrhage	1.05 (1.01;1.10)
Personal psychiatric history - Acute C-section	1.16 (1.12;1.21)
Personal psychiatric history - Preterm birth	1.08 (1.01;1.16)

* Association between psychiatric history and complications were adjusted for parity, calendar year, maternal age, educational level, and country of origin.

eTable 3. Logistic regressions estimating OR (95% CI) for the mediator stratified by the exposure

	PPD symptoms		PPD diagnosis	
	PH: No*	PH: Yes*	PH: No*	PH: Yes*
Any complications	1.20 (1.14;1.26)	1.22 (1.00;1.49)	1.09 (1.02;1.17)	1.15 (0.97;1.36)
Preeclampsia/eclampsia	1.20 (1.06;1.35)	1.13 (0.69;1.84)	1.09 (0.93;1.28)	0.95 (0.62;1.45)
Gestational hypertension	1.17 (1.02;1.35)	0.92 (0.49;1.72)	0.82 (0.67;1.01)	0.84 (0.50;1.42)
Gestational diabetes	1.19 (1.07;1.32)	1.40 (0.95;2.04)	1.02 (0.89;1.16)	1.00 (0.72;1.39)
Hyperemesis gravidarum	1.52 (1.33;1.74)	1.93 (1.22;3.08)	1.25 (1.07;2.45)	1.60 (1.15;2.23)
Postpartum hemorrhage	1.05 (0.97;1.15)	1.03 (0.73;1.46)	1.03 (0.92;1.16)	1.06 (0.80;1.41)
Acute C-section	1.16 (1.09;1.24)	1.21 (0.95;1.56)	1.15 (1.06;1.25)	1.18 (0.96;1.45)
Preterm birth	1.37 (1.22;1.55)	1.66 (1.08;2.56)	1.27 (1.08;1.50)	0.98 (0.63;1.52)

*Adjusted for parity, calendar year, maternal age, educational level, and origin of birth.
Abbreviation: PH, personal psychiatric history

eTable 4. Mediation analysis estimating OR (95% CI) and the proportion mediated for specific complications

	PPD symptoms			
	Direct effect	Indirect effect	Total effect	Proportion mediated (95% CI)*
Preeclampsia/eclampsia	1.07 (1.07;1.08)	1.00 (1.00;1.00)	1.07 (1.07;1.08)	0.07% (0.06%;0.18%)
Gestational hypertension	1.07 (1.07;1.08)	1.00 (1.00;1.00)	1.07 (1.07;1.08)	0.01% (0.00%;0.07%)
Gestational diabetes	1.07 (1.07;1.08)	1.00 (1.00;1.00)	1.07 (1.07;1.08)	0.13% (0.08%;0.25%)
Hyperemesis gravidarum	1.07 (1.07;1.07)	1.00 (1.00;1.00)	1.07 (1.07;1.07)	0.68% (0.52%;1.20%)
Postpartum hemorrhage	1.07 (1.07;1.07)	1.00 (1.00;1.00)	1.07 (1.07;1.07)	0.01% (0.00%;0.07%)
Acute C-section	1.07 (1.07;1.07)	1.00 (1.00;1.00)	1.07 (1.07;1.08)	0.34% (0.22%;0.45%)
Preterm birth	1.07 (1.07;1.07)	1.00 (1.00;1.00)	1.07 (1.07;1.07)	0.04% (0.01%;0.13%)

*Negative values have been set to zero

	PPD diagnosis			
	Direct effect	Indirect effect	Total effect	Proportion mediated (95% CI)*
Preeclampsia/eclampsia	1.02 (1.01;1.02)	1.00 (1.00;1.00)	1.02 (1.01;1.02)	0.02% (0.00%;0.08%)
Gestational hypertension	1.01 (1.01;1.02)	1.00 (1.00;1.00)	1.01 (1.01;1.02)	0.00% (0.00%;0.05%)
Gestational diabetes	1.01 (1.01;1.02)	1.00 (1.00;1.00)	1.01 (1.01;1.02)	0.15% (0.00%;0.27%)
Hyperemesis gravidarum	1.02 (1.01;1.02)	1.00 (1.00;1.00)	1.02 (1.01;1.02)	0.68% (0.52%;1.12%)
Postpartum hemorrhage	1.01 (1.01;1.02)	1.00 (1.00;1.00)	1.01 (1.01;1.02)	0.01% (0.00%;0.07%)
Acute C-section	1.02 (1.01;1.02)	1.00 (1.00;1.00)	1.02 (1.01;1.02)	0.27% (0.00%;0.45%)
Preterm birth	1.02 (1.01;1.02)	1.00 (1.00;1.00)	1.02 (1.01;1.02)	0.03% (0.01%;0.09%)

*Negative values have been set to zero

eTable 5. Mediation analysis estimating OR (95% CI) and the proportion mediated stratified by timing of the exposure (recent and past)

	PPD symptoms			
Mediator	Direct effect	Indirect effect	Total effect	Proportion mediated*
Recent				
Any complication	1.10 (1.09;1.11)	1.00 (1.00;1.00)	1.10 (1.10;1.11)	0.62% (0.44%;0.82%)
Preeclampsia/eclampsia	1.10 (1.09;1.11)	1.00 (1.00;1.00)	1.10 (1.09;1.11)	0.08% (0.01%;0.15%)
Gestational hypertension	1.10 (1.09;1.11)	1.00 (1.00;1.00)	1.10 (1.09;1.11)	0.01% (0.00%;0.05%)
Gestational diabetes	1.10 (1.09;1.11)	1.00 (1.00;1.00)	1.10 (1.09;1.11)	0.14% (0.03%;0.28%)
Hyperemesis gravidarum	1.10 (1.09;1.11)	1.00 (1.00;1.00)	1.10 (1.09;1.11)	0.63% (0.47%;0.96%)
Postpartum hemorrhage	1.10 (1.09;1.11)	1.00 (1.00;1.00)	1.10 (1.09;1.11)	0.00% (0.00%;0.04%)
C-section	1.10 (1.09;1.11)	1.00 (1.00;1.00)	1.10 (1.09;1.11)	0.38% (0.23%;0.56%)
Preterm birth	1.10 (1.09;1.11)	1.00 (1.00;1.00)	1.10 (1.09;1.11)	0.06% (0.00%;0.16%)
Past				
Any complication	1.05 (1.05,1.06)	1.00 (1.00;1.00)	1.06 (1.05,1.06)	0.73% (0.53%;0.93%)
Preeclampsia/eclampsia	1.06 (1.05,1.06)	1.00 (1.00;1.00)	1.06 (1.05,1.06)	0.07% (0.01%;0.16%)
Gestational hypertension	1.06 (1.05,1.06)	1.00 (1.00;1.00)	1.06 (1.05,1.06)	0.01% (0.00%;0.08%)
Gestational diabetes	1.06 (1.05,1.06)	1.00 (1.00;1.00)	1.06 (1.05,1.06)	0.14% (0.05%;0.27%)
Hyperemesis gravidarum	1.06 (1.05,1.06)	1.00 (1.00;1.00)	1.06 (1.05,1.06)	0.68% (0.39%;0.97%)
Postpartum hemorrhage	1.06 (1.05,1.06)	1.00 (1.00;1.00)	1.06 (1.05,1.06)	0.04% (0.00%;0.11%)
C-section	1.06 (1.05,1.06)	1.00 (1.00;1.00)	1.05 (1.05,1.06)	0.36% (0.18%;0.54%)
Preterm birth	1.06 (1.05,1.06)	1.00 (1.00;1.00)	1.05 (1.05,1.06)	0.11% (0.00%;0.25%)

*Negative values have been set to zero

	PPD diagnosis			
Mediator	Direct effect	Indirect effect	Total effect	Proportion mediated*
Recent				
Any complication	1.03 (1.03;1.04)	1.00 (1.00;1.00)	1.03 (1.03;1.04)	0.40% (0.04%;0.81%)
Preeclampsia/eclampsia	1.03 (1.03;1.04)	1.00 (1.00;1.00)	1.03 (1.03;1.04)	0.00% (0.00%;0.14%)
Gestational hypertension	1.03 (1.03;1.04)	1.00 (1.00;1.00)	1.03 (1.03;1.04)	0.00% (0.00%;0.07%)
Gestational diabetes	1.03 (1.03;1.04)	1.00 (1.00;1.00)	1.03 (1.03;1.04)	0.10% (0.00%;0.34%)
Hyperemesis gravidarum	1.03 (1.03;1.04)	1.00 (1.00;1.00)	1.03 (1.03;1.04)	0.71% (0.31%;1.13%)
Postpartum hemorrhage	1.03 (1.03;1.04)	1.00 (1.00;1.00)	1.03 (1.03;1.04)	0.00% (0.00%;0.04%)
C-section	1.03 (1.03;1.04)	1.00 (1.00;1.00)	1.03 (1.03;1.04)	0.26% (0.00%;0.56%)
Preterm birth	1.03 (1.03;1.04)	1.00 (1.00;1.00)	1.03 (1.03;1.04)	0.02% (0.00%;0.13%)
Past				
Any complication	1.01 (1.00;1.01)	1.00 (1.00;1.00)	1.01 (1.00;1.01)	0.61% (0.14%;1.09%)
Preeclampsia/eclampsia	1.01 (1.00;1.01)	1.00 (1.00;1.00)	1.01 (1.00;1.01)	0.00% (0.00%;0.14%)
Gestational hypertension	1.01 (1.00;1.01)	1.00 (1.00;1.00)	1.01 (1.00;1.01)	0.00% (0.00%;0.11%)
Gestational diabetes	1.01 (1.00;1.01)	1.00 (1.00;1.00)	1.01 (1.00;1.01)	0.11% (0.00%;0.51%)
Hyperemesis gravidarum	1.01 (1.00;1.01)	1.00 (1.00;1.00)	1.01 (1.00;1.01)	0.89% (0.31%;1.53%)
Postpartum hemorrhage	1.01 (1.00;1.01)	1.00 (1.00;1.00)	1.01 (1.00;1.01)	0.05% (0.00%;0.30%)
C-section	1.01 (1.00;1.01)	1.00 (1.00;1.00)	1.01 (1.00;1.01)	0.30% (0.01%;0.67%)
Preterm birth	1.01 (1.00;1.01)	1.00 (1.00;1.00)	1.01 (1.00;1.01)	0.05% (0.00%;0.24%)

*Negative values have been set to zero

eTable 6. Sensitivity analysis of mediation analysis restricted to one random birth per mother in HOPE

	PPD symptoms			
Mediator	Direct effect	Indirect effect	Total effect	Proportion mediated (95% CI)
Any complication	1.07 (1.07;1.07)	1.00 (1.00;1.00)	1.07 (1.07;1.07)	0.91% (0.54%;1.04%)
Preeclampsia/eclampsia	1.07 (1.07;1.08)	1.00 (1.00;1.00)	1.07 (1.07;1.08)	0.11% (0.04%;0.15%)
Gestational hypertension	1.07 (1.07;1.08)	1.00 (1.00;1.00)	1.07 (1.07;1.08)	0.03% (0.00%;0.07%)
Gestational diabetes	1.07 (1.07;1.08)	1.00 (1.00;1.00)	1.07 (1.07;1.08)	0.14% (0.08%;0.26%)
Hyperemesis gravidarum	1.07 (1.07;1.07)	1.00 (1.00;1.00)	1.07 (1.07;1.07)	0.81% (0.48%;1.08%)
Postpartum hemorrhage	1.07 (1.07;1.08)	1.00 (1.00;1.00)	1.07 (1.07;1.08)	0.03% (0.00%;0.07%)
C-section	1.07 (1.07;1.08)	1.00 (1.00;1.00)	1.07 (1.07;1.08)	0.48% (0.29%;0.66%)
Preterm birth	1.07 (1.07;1.08)	1.00 (1.00;1.00)	1.07 (1.07;1.08)	0.13% (0.02%;0.18%)

	PPD diagnosis			
Mediator	Direct effect	Indirect effect	Total effect	Proportion mediated
Any complication	1.02 (1.01;1.02)	1.00 (1.00;1.00)	1.02 (1.01;1.02)	0.48% (0.13%;1.70%)
Preeclampsia/eclampsia	1.02 (1.01;1.02)	1.00 (1.00;1.00)	1.02 (1.01;1.02)	0.00% (0.00%;0.07%)
Gestational hypertension	1.02 (1.01;1.02)	1.00 (1.00;1.00)	1.02 (1.01;1.02)	0.00% (0.00%;0.03%)
Gestational diabetes	1.02 (1.01;1.02)	1.00 (1.00;1.00)	1.02 (1.01;1.02)	0.19% (0.00%;0.27%)
Hyperemesis gravidarum	1.02 (1.01;1.02)	1.00 (1.00;1.00)	1.02 (1.01;1.02)	0.76% (0.47%;1.28%)
Postpartum hemorrhage	1.02 (1.01;1.02)	1.00 (1.00;1.00)	1.02 (1.01;1.02)	0.04% (0.00%;0.09%)
C-section	1.02 (1.01;1.02)	1.00 (1.00;1.00)	1.02 (1.01;1.02)	0.32% (0.21%;0.64%)
Preterm birth	1.02 (1.01;1.02)	1.00 (1.00;1.00)	1.02 (1.01;1.02)	0.04% (0.00%;0.07%)

eTable 7. Sensitivity analysis of mediation analysis with exclusion of women with no prior psychiatric history who receive a psychiatric diagnosis or psychotropic medication from conception until delivery

	PPD symptoms			
Mediator	Direct effect	Indirect effect	Total effect	Proportion mediated (95% CI)
Any complication	1.07 (1.07;1.07)	1.00 (1.00;1.00)	1.07 (1.07;1.07)	0.65% (0.58%;0.82%)
Preeclampsia/eclampsia	1.07 (1.07;1.07)	1.00 (1.00;1.00)	1.07 (1.07;1.07)	0.09% (0.04%;0.13%)
Gestational hypertension	1.07 (1.07;1.08)	1.00 (1.00;1.00)	1.07 (1.07;1.08)	0.01% (0.00%;0.06%)
Gestational diabetes	1.07 (1.07;1.07)	1.00 (1.00;1.00)	1.07 (1.07;1.07)	0.16% (0.10%;0.24%)
Hyperemesis gravidarum	1.07 (1.07;1.08)	1.00 (1.00;1.00)	1.07 (1.07;1.08)	0.70% (0.54%;1.02%)
Postpartum hemorrhage	1.07 (1.07;1.07)	1.00 (1.00;1.00)	1.07 (1.07;1.07)	0.02% (0.00%;0.05%)
C-section	1.07 (1.07;1.07)	1.00 (1.00;1.00)	1.07 (1.07;1.07)	0.40% (0.20%;0.46%)
Preterm birth	1.07 (1.07;1.07)	1.00 (1.00;1.00)	1.07 (1.07;1.07)	0.11% (0.01%;0.23%)

	PPD diagnosis			
Mediator	Direct effect	Indirect effect	Total effect	Proportion mediated
Any complication	1.02 (1.01;1.02)	1.00 (1.00;1.00)	1.02 (1.01;1.02)	0.44% (0.07%;0.66%)
Preeclampsia/eclampsia	1.02 (1.01;1.02)	1.00 (1.00;1.00)	1.02 (1.01;1.02)	0.01% (0.00%;0.16%)
Gestational hypertension	1.01 (1.01;1.02)	1.00 (1.00;1.00)	1.01 (1.01;1.02)	0.00% (0.00%;0.06%)
Gestational diabetes	1.01 (1.01;1.02)	1.00 (1.00;1.00)	1.01 (1.01;1.02)	0.08% (0.00%;0.27%)
Hyperemesis gravidarum	1.01 (1.01;1.02)	1.00 (1.00;1.00)	1.01 (1.01;1.02)	0.63% (0.52%;1.06%)
Postpartum hemorrhage	1.01 (1.01;1.02)	1.00 (1.00;1.00)	1.01 (1.01;1.02)	0.01% (0.00%;0.03%)
C-section	1.01 (1.01;1.02)	1.00 (1.00;1.00)	1.01 (1.01;1.02)	0.28% (0.12%;0.61%)
Preterm birth	1.01 (1.01;1.02)	1.00 (1.00;1.00)	1.01 (1.01;1.02)	0.03% (0.00%;0.08%)

eTable 8. Sensitivity analysis estimating the E-value for the indirect effect

Mediators	PPD symptoms Estimate	PPD diagnosis Estimate
Any complication	1.00	1.00
Preeclampsia/eclampsia	1.00	1.00
Gestational hypertension	1.00	1.00
Gestational diabetes	1.00	1.00
Hyperemesis gravidarum	1.03	1.00
Postpartum hemorrhage	1.00	1.00
C-section	1.00	1.00
Preterm birth	1.00	1.00

*Lowest possible E-value is 1 (i.e., no unmeasured confounding is needed to explain away the observed association)

eTable 9. Sensitivity analysis estimating the E-value for the direct effect

	PPD symptoms		PPD diagnosis	
Mediators	Estimate	Lower CI	Estimate	Lower CI
Any complication	1.34	1.33	1.13	1.13
Preeclampsia/eclampsia	1.35	1.34	1.14	1.13
Gestational hypertension	1.35	1.34	1.13	1.13
Gestational diabetes	1.35	1.34	1.13	1.13
Hyperemesis gravidarum	1.34	1.34	1.14	1.13
Postpartum hemorrhage	1.34	1.34	1.13	1.12
C-section	1.34	1.33	1.14	1.13
Preterm birth	1.34	1.33	1.14	1.13

[*] Lowest possible E-value is 1 (i.e., no unmeasured confounding is needed to explain away the observed association)
